# Around the Hospitals

**Published:** 1893-07-15

**Authors:** 


### Around the Hospitals.

Notice to the Managers of Institutions.—Special space is now reserved for the insertion of notices of hospital meetings and festivals. The Editor requests that all notices may reach The Hospital Office, 428, Strand, at least one week before the date of each meeting.

**The Edinburgh Royal Infirmary.**—The number of in-patients treated at this institution during the past year was 8,637. More than half this number were received from the country. Unlike the experience of many a city hospital, this fact is thoroughly recognized in connection with the Edinburgh Infirmary, and more than half the contributions received are from the country also. The total income for 1892 showed a slight decrease on that of the previous year. The diminution shows itself in donations and legacies. Individuals and firms in Edinburgh have not contributed so well as formerly, but public works and establishments show a substantial increase, a gratifying fact which reveals the appreciation in which the institution is held by the working classes. Church collections have not been so satisfactory, and this is a source to which the infirmary may well look for the increasing amount of expenditure necessary to provide for a larger number of beds and the necessary wear and tear of furniture and fittings. The expenditure for the year amounted to £41,970, over £1,500 in excess of that of the previous year. Part of the increase of expenditure has been in the nursing departments, a fact which the public are not likely to regret, seeing that Edinburgh sets an example in its training and care of its nurses which it is hard to beat throughout Great Britain. The Convalescent Home has been increased by two wings, containing forty beds, through the munificent bequest of Mr. Nasmyth, of Penhurst. The managers are fortunate in being able to entrust the care of the Home to the hands of an excellent Lady Superintendent, Miss Fergusson. The Infirmary lost two excellent officers last year in the persons of Mr. Fasson, the late Superintendent, and Dr. A. Keiller, Consulting Physician. There is a feature in the annual report of the Edinburgh Royal Infirmary to which we should like to draw the attention of city institutions. This is the list of contributors names given under the heading of the name of the street in which they live. It is thus possible to ascertain at a glance the extent to which a community is represented in the support of a charity, and such a list is of great assistance to those collecting.

**The Seaman's Hospital.**—The work of this hospital and its auxilliaries continues to increase. Last year 8,578 in-patients were treated at the parent institution at Greenwich, 3,709 at the Branch Hospital, 1,105 at the Gravesend Dispensary, and 3,043 at the Dispensary in the East India Dock Road. Funds, unfortunately, do not show a proportionate increase, and the adverse balance at the end of the year amounted to £2,102. This is slightly less than the deficiency during the previous year, however. The hospital is economically managed, and the cost per bed compares favourably with that of hospitals of a similar size in the metropolis. The hospital confers international benefits, and this fact is recognised by most of the crowned heads of Europe, who are subscribers to the hospital. The branch of the Seaman's Hospital Society, which occupies itself with assisting disabled sailors on leaving the hospital, is not the least useful portions of its work. Through this agency sailors are enabled to reach their homes and to go to ports from whence they can ship. The Steam Navigation Company usefully co-operates with the society in this respect. The Marine service attracts very general sympathy, and were the manyways in which the public could help the hospital's work more widely understood, we feel sure numbers of willing helpers would be forthcoming.

